# Deep divergences and extensive phylogeographic structure in a clade of lowland tropical salamanders

**DOI:** 10.1186/1471-2148-12-255

**Published:** 2012-12-29

**Authors:** Sean M Rovito, Gabriela Parra-Olea, Carlos R Vásquez-Almazán, Roberto Luna-Reyes, David B Wake

**Affiliations:** 1 Museum of Vertebrate Zoology, 3101 Valley Life Sciences Building, University of California, Berkeley, CA, 94720-3160, USA; 2 Instituto de Biología, Universidad Nacional Autónoma de México, AP 70–153, Circuito Exterior, Ciudad Universitaria, CP 04310, México, D.F., México; 3 Museo de Historia Natural, Escuela de Biología, Universidad de San Carlos, Calle Mariscal Cruz 1-56, Zona 10, Ciudad de Guatemala, Guatemala; 4 Coordinación Técnica de Investigación, Secretaría de Medio Ambiente e Historia Natural, Av. de los Hombres Ilustres s/n, Fraccionamiento Francisco I. Madero, Tuxtla Gutiérrez, CP 29000, Chiapas, México; 5 Museum of Vertebrate Zoology and Department of Integrative Biology, 3101 Valley Life Sciences Building, University of California, Berkeley, CA, 94720-3160, USA

**Keywords:** Salamander, Phylogeography, Mesoamerica, Isthmus of Tehuantepec, Biogeography, *Bolitoglossa*

## Abstract

**Background:**

The complex geological history of Mesoamerica provides the opportunity to study the impact of multiple biogeographic barriers on population differentiation. We examine phylogeographic patterns in a clade of lowland salamanders (*Bolitoglossa* subgenus *Nanotriton*) using two mitochondrial genes and one nuclear gene. We use several phylogeographic analyses to infer the history of this clade and test hypotheses regarding the geographic origin of species and location of genetic breaks within species. We compare our results to those for other taxa to determine if historical events impacted different species in a similar manner.

**Results:**

Deep genetic divergence between species indicates that they are relatively old, and two of the three widespread species show strong phylogeographic structure. Comparison of mtDNA and nuclear gene trees shows no evidence of hybridization or introgression between species. Isolated populations of *Bolitoglossa rufescens* from Los Tuxtlas region constitute a separate lineage based on molecular data and morphology, and divergence between Los Tuxtlas and other areas appears to predate the arrival of *B. rufescens* in other areas west of the Isthmus of Tehuantepec. The Isthmus appears responsible for Pliocene vicariance within *B. rufescens*, as has been shown for other taxa. The Motagua-Polochic fault system does not appear to have caused population vicariance, unlike in other systems.

**Conclusions:**

Species of *Nanotriton* have responded to some major geological events in the same manner as other taxa, particularly in the case of the Isthmus of Tehuantepec. The deep divergence of the Los Tuxtlas populations of *B. rufescens* from other populations highlights the contribution of this volcanic system to patterns of regional endemism, and morphological differences observed in the Los Tuxtlas populations suggests that they may represent an undescribed species of *Bolitoglossa*. The absence of phylogeographic structure in *B. nympha*, in contrast to the other widespread species in the subgenus, may be due to historical forest contraction and more recent range expansion in the region. Phylogeographic data provide substantial insight into the evolutionary history of these morphologically similar species of salamanders, and contribute to our understanding of factors that have generated the high biodiversity of Mesoamerica.

## Background

Phylogeography and molecular systematics have been of great utility for delimiting the species boundaries of morphologically cryptic taxa [1-3] and understanding the origins of diversity both at and below the species level [4-7]. Within taxa, high genetic diversity in a small region often indicates long-term historical persistence in the face of environmental change, while genetic breaks between populations suggest a more complex demographic history involving periods of isolation or restricted gene flow. Climatic barriers or geological features are often associated with such phylogeographic breaks and can be hypothesized to have caused population isolation. Given that allopatric divergence is hypothesized to be the prevailing geographic mode of speciation for most animal taxa [8], understanding which barriers have led to divergence within species should elucidate the impact of such barriers on species diversity over longer timescales. An improved understanding of both species boundaries and phylogeographic structure enables tests of hypotheses related to the geographic origin of clades, connecting their divergence to regional processes, and facilitates comparisons of patterns of lineage divergence across taxa.

Understanding the historical factors promoting population divergence and species formation is of particular interest in areas of high species diversity, such as humid tropical regions. Here we investigate how the complex geological history of southern Mexico and Nuclear Central America, the area between the Isthmus of Tehuantepec and the Nicaraguan Depression [9], relates to population divergence in a group of morphologically similar plethodontid salamanders. We interpret their divergence history in the light of patterns seen in other taxa in order to understand how regional biogeography may have influenced current patterns of species diversity and faunistic relationships between subregions.

Salamanders have often been shown to have historical signatures of geological or climatic changes in their geographic patterns of genetic variation [10-12], due to their short dispersal distances [13] and environmental sensitivity [14]. Most species of Neotropical salamanders, however, are characterized by small range sizes, and often are known from only a single mountain range [15-17], precluding studies of population differentiation at a regional scale. This tendency toward small range size could be a direct result of stronger population isolation in the tropics, leading to higher rates of allopatric speciation and thus producing many species with small geographic ranges [18,19]. The few wide-ranging tropical salamanders offer the chance to study species of low dispersal ability that are distributed across major geographic barriers, and allow us to understand the processes that may have been important in generating the high diversity seen in the tropical salamanders as a whole.

**Figure 1:**
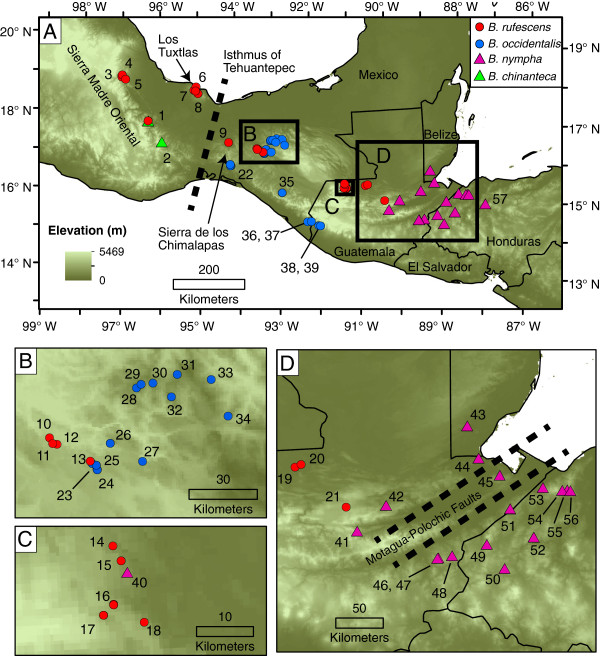
**Samples used in phylogenetic analyses.** Red circles: *Bolitoglossa rufescens*; blue circles: *B. occidentalis*; pink triangles: *B. nympha*; green triangles: *B. chinanteca*. Numbers correspond to localities listed in Table 1. Species assignment based on phylogenetic analyses (see Results).

Our study focuses on species of *Bolitoglossa* subgenus *Nanotriton*, which has only four described species, yet is distributed throughout a wide area of Mesoamerica [20]. The species of this subgenus span multiple biogeographic boundaries in Mesoamerica, including the Isthmus of Tehuantepec, which appears to have restricted dispersal of mesic-adapted species [21-23], while acting as a corridor for arid-adapted species [24]. Species in the subgenus occur in multiple geological regions, including the eastern terminus of the Trans-Mexican Volcanic Belt (TMVB) and Los Tuxtlas region in Veracruz, the Northern highlands of Oaxaca, the Sierra de los Chimalapas, Mexico and on both the Pacific and Caribbean sides of Chiapas, Mexico and Guatemala in Nuclear Central America (Figure 1). *Bolitoglossa rufescens*, the most widely distributed species in the subgenus, ranges across the Isthmus of Tehuantepec, making it one of only three salamander species to occur on both sides of this major barrier [25,26]. As presently known, *B. rufescens* also occurs on both sides of the Motagua-Polochic fault system, an important barrier for many other taxa in Central America [21,22].

Although species of *Nanotriton* generally occur only in mid- to low elevations, populations are known from up to 2000 m elevation and are not found in dry forest, savannah, or other low-elevation subhumid habitats. Many populations are currently associated with banana plantations and other anthropogenic habitats adjacent to forest, but salamanders can also be found in forested habitats. Because of these associations, species of *Nanotriton*, like higher elevation salamanders, could be used to test for effects of historical forest fragmentation or expansion, in addition to examining effects of geological barriers such as major mountain ranges or fault zones.

In at least two localities, two species of the subgenus are found in sympatry [27,28] and another instance of near-sympatry is known [29], despite overall morphological similarity of these species that might be expected to limit their co-occurrence [27]. The only obvious character separating two of the species (*Bolitoglossa chinanteca* and *B. occidentalis*) from the other two (*B. nympha* and *B. rufescens*) is the presence or absence of maxillary teeth, and even this character is variable within *B. rufescens*[29]. This group of salamanders exhibits little variation in morphology, possibly because of their generally paedomorphic state; development of features such as digits or skull bones that distinguish other species of salamanders is truncated, leading to a reduction or absence of these elements [30,31]. Because of this high similarity in external appearance, genetic markers provide one of the best tools to delimit these species’ geographical limits, an essential endeavor for understanding the biogeographic history of the group. The use of both mtDNA and nuclear markers also allows us to test for a signature of introgression across species boundaries, with finer-scale sampling in possible areas of contact identified by analyses of allozymes [29].

In this study, we use both mitochondrial (mtDNA) and nuclear DNA (nDNA) to investigate phylogeographic structure within species of *Nanotriton* as well as phylogenetic relationships between described species. We compare patterns of mtDNA and nDNA to look for discordance that might indicate introgression across species boundaries, especially near previously identified possible contact zones, and to identify hybrids or signatures of past population admixture. We use a likelihood-based phylogeographic history estimation method (Phylomapper [32]) to test hypotheses of the geographic location of the origins of clades. In particular, we examine in which biogeographic region (southern Mexico vs. Central America) and in which mountain range or subregion species originated. We examine the impact of major biogeographic boundaries known from other taxa on the phylogeographic patterns within these salamander species, and compare patterns across these boundaries to those seen in other taxa.

## Results

**Figure 2:**
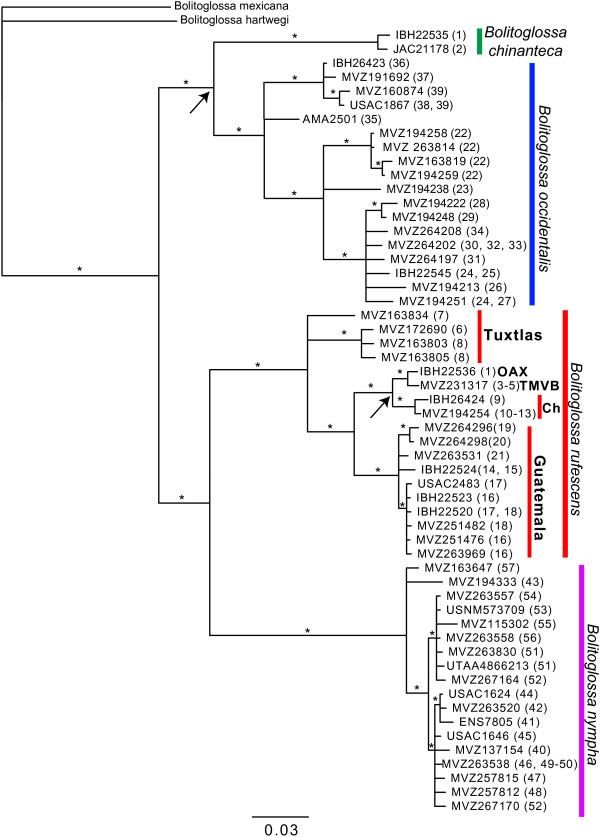
**Mitochondrial gene tree from RAxML analysis of *16S *and *cytb *sequence data.** Asterisks indicate branches with bootstrap proportions >70 for ML analysis and posterior propabilities >95 for Bayesian analysis. Numbers in parentheses after voucher numbers refer to all sampled localities (from Table 1) where haplotype was found. Geographic areas are shown for samples of *Bolitoglossa rufescens*. Abbreviations: Ch–Chiapas; TMVB–eastern terminus of Trans-Mexican Volcanic Belt; OAX–highlands of northern Oaxaca. Arrows indicate nodes whose daughter lineages are separated by the Isthmus of Tehuantepec.

Phylogenetic analyses reveal deep divergences between species, as well as a high degree of phylogeographic structure within species. The mtDNA results (Figure 2) strongly support the monophyly of *Nanotriton* (likelihood bootstrap proportion [BS]=100, posterior probability [PP]=1.0), as well as that of all four members of the group: *Bolitoglossa occidentalis* (BS=99, PP=1.0), *B. chinanteca* (BS=100, PP=1.0), *B. nympha* (BS=100, PP=1.0) and *B. rufescens* (BS=100, PP=1.0). *B. occidentalis* and *B*. *chinanteca* are strongly supported as sister taxa (BS=99, PP=1.0), as are *B. nympha* and *B. rufescens* (BS=96, PP=0.99).

Within species, markedly different patterns of phylogeographic structure emerge. *Bolitoglossa chinanteca* shows little divergence in *16S* sequence between the only known localities (GTR distance=0.004 substitutions/site). *Bolitoglossa occidentalis* shows several strongly supported lineages (BS>70, PP>95) corresponding to populations from 1) the Pacific coast of Chiapas and Guatemala, 2) a single population from the eastern side (Caribbean-draining) side of the Sierra Madre de Chiapas (Locality 35; Figure 1, Table 1), 3) a single population from Cerro Baúl in the isolated Sierra de los Chimalapas (Locality 22), and 4) various populations from other areas of northern and central Chiapas (Localities 23–34). Maximum GTR distances between samples of *B. occidentalis* are large (0.043 for *16S* between localities 39 and 28, 0.10 for *cytb* between localities 22 and 37); GTR distances between individuals from all localities for each gene are given in Additional files 1, 2, 3: Tables S2, S3, S4. Our samples from the Sierra de los Chimalapas (locality 22; Figure 1) are from near the type locality of *Bolitoglossa bilineata*[33], synonymized with *B. occidentalis* by Wake and Brame [34]. Locality 22 is nested within *B. occidentalis* in our mtDNA gene tree*.*

**Table 1:** **Populations of*Bolitoglossa*(*Nanotriton*) used in phylogenetic analyses**

**Locality number**	**Species**	**Country: State/Province**	**Locality**
1	*B. chinanteca, B. rufescens*	Mexico: Oaxaca	9.2 km S of Valle Nacional on Hwy 175
2	*B. chinanteca*	Mexico: Oaxaca	Coconales-Zacatepec highway, Sierra Mixe
3	*B. rufescens*	Mexico: Veracruz	Cerro Chicahuaxtla, Cuautlapan
4	*B. rufescens*	Mexico: Veracruz	Fortín de las Flores
5	*B. rufescens*	Mexico: Veracruz	Coetzala, 8.9 km S of Amatlán
6	*B. rufescens*	Mexico: Veracruz	Playa Escondida, 30 km NNE Catemaco
7	*B. rufescens*	Mexico: Veracruz	9.2 km NE of Catemaco
8	*B. rufescens*	Mexico: Veracruz	Lake Catemaco, 2.5 km SE Coyame
9	*B. rufescens*	Mexico: Oaxaca	1.5 km SE of La Fortaleza
10	*B. rufescens*	Mexico: Chiapas	10 km NW Ocuilapa, Ocozocautla
11	*B. rufescens*	Mexico: Chiapas	15 km N Ocozocuautla
12	*B. rufescens*	Mexico: Chiapas	26.5 km N Ocozocuautla
13	*B. rufescens*	Mexico: Chiapas	12.4 km W Berriozabal
14	*B. rufescens*	Guatemala: Huehuetenango	Siglo Veinte Ermin, Barillas
15	*B. rufescens*	Guatemala: Huehuetenango	Las Victorias Chancolin, Barillas
16	*B. rufescens*	Guatemala: Huehuetenango	Palmiras de Chiblac, Barillas
17	*B. rufescens*	Guatemala: Huehuetenango	El Valle, 4.5 km N of RN 9 at Aldea La Concepción
18	*B. rufescens*	Guatemala: Huehuetenango	San Ramon, Barillas
19	*B. rufescens*	Guatemala: Alta Verapaz	western border of Parque Nacional Laguna Lachua
20	*B. rufescens*	Guatemala: Alta Verapaz	Parque Nacional Laguna Lachua
21	*B. rufescens*	Guatemala: Alta Verapaz	Finca Cuxmax, San Pedro Carchá
22	*B. occidentalis*	Mexico: Chiapas	Cerro Baúl
23	*B. occidentalis*	Mexico: Chiapas	11.4 km NW Berriozabal
24	*B. occidentalis*	Mexico: Chiapas	Vista Hermosa, 7.5 km N Berriozabal
25	*B. occidentalis*	Mexico: Chiapas	Cuhumbac, 10.4 km N Berriozabal
26	*B. occidentalis*	Mexico: Chiapas	11.2 km N San Fernando, Tuxtla Gutiérrez
27	*B. occidentalis*	Mexico: Chiapas	W San Fernando, 13 km N Tuxtla Gutiérrez
28	*B. occidentalis*	Mexico: Chiapas	15.3 km ENE Copainala
29	*B. occidentalis*	Mexico: Chiapas	9 km ENE Coapilla
30	*B. occidentalis*	Mexico: Chiapas	2.7 km W Pantepec
31	*B. occidentalis*	Mexico: Chiapas	Puerto del Viento, Pueblo Nuevo Solistahuacán
32	*B. occidentalis*	Mexico: Chiapas	Julian Grijales, W of Pueblo Nuevo Solistahuacán
33	*B. occidentalis*	Mexico: Chiapas	W of Rayon
34	*B. occidentalis*	Mexico: Chiapas	SE of Puerto Cate
35	*B. occidentalis*	Mexico: Chiapas	Finca Prusia
36	*B. occidentalis*	Mexico: Chiapas	14 km N Tapachula on road to Finca Nueva Alemania
37	*B. occidentalis*	Mexico: Chiapas	7.5 km N Cacahoatán
38	*B. occidentalis*	Guatemala: San Marcos	Finca Santa Julia
39	*B. occidentalis*	Guatemala: San Marcos	2 km S San Rafael Pie de la Cuesta
40	*B. nympha*	Guatemala: Huehuetenango	Chancolín
41	*B. nympha*	Guatemala: Baja Verapaz	Finca Sabó, Purulhá
42	*B. nympha*	Guatemala: Alta Verapaz	Finca el Volcán, Senahú
43	*B. nympha*	Belize: Toledo	Blue Creek National Park
44	*B. nympha*	Guatemala: Izabal	Cerro Sarstún, Lívingston
45	*B. nympha*	Guatemala: Izabal	Las Escobas, Cerro San Gil
46	*B. nympha*	Guatemala: Zacapa	Finca la Bendición, Pinalito
47	*B. nympha*	Guatemala: Zacapa	Finca las Granadillas, Pinalito
48	*B. nympha*	Guatemala: Zacapa	5.2 km SE La Unión
49	*B. nympha*	Honduras: Copan	El Limón, Sierra del Espíritu Santo
50	*B. nympha*	Honduras: Copan	Santa Rosa de Copan
51	*B. nympha*	Guatemala: Izabal	Finca la Firmeza, Sierra Caral
52	*B. nympha*	Honduras: Santa Barbara	Montaña de Joconales
53	*B. nympha*	Honduras: Cortés	Santa Teresita
54	*B. nympha*	Honduras: Cortés	Aldea Buenos Aires
55	*B. nympha*	Honduras: Cortés	Sierra del Espíritu Santo, W San Pedro Sula
56	*B. nympha*	Honduras: Cortés	11 km W of CA-5 at Hospital Cemesa, San Pedro Sula
57	*B. nympha*	Honduras: Yoro	38.6 km NE Santa Rita

*Bolitoglossa rufescens* comprises several well-supported, divergent lineages. Two groups of populations from the Los Tuxtlas region form a basal polytomy with all remaining populations. Samples from either side of the Isthmus of Tehuantepec are not reciprocally monophyletic; those from the eastern end of the TMVB in Veracruz and northern highlands of Oaxaca are in a clade with samples from Chiapas and the Sierra de los Chimalapas (Figure 2). Yet another clade of *B. rufescens* consists of samples from northwestern Guatemala.

Although *B. nympha* was described only from the type locality (locality 50) in Guatemala [35], Rovito et al. [27] hypothesized that populations from eastern Guatemala and western Honduras could be assigned to this species based on morphological data as well as allozyme results from Larson [29]. Campbell et al. [35] also stated that a specimen from Belize might be assignable to *B. nympha* based on a published *cytb* sequence. The mtDNA gene tree shows a deep divergence between populations formerly assigned to *B. rufescens* from Mexico and northwestern Guatemala and those from eastern Guatemala, Belize, and Honduras (Figures 1, 2). Given that the latter group of populations includes the type locality of *B. nympha*, we assign all these populations to that species, which is now known from a much broader geographic area than in the original description.

**Figure 3:**
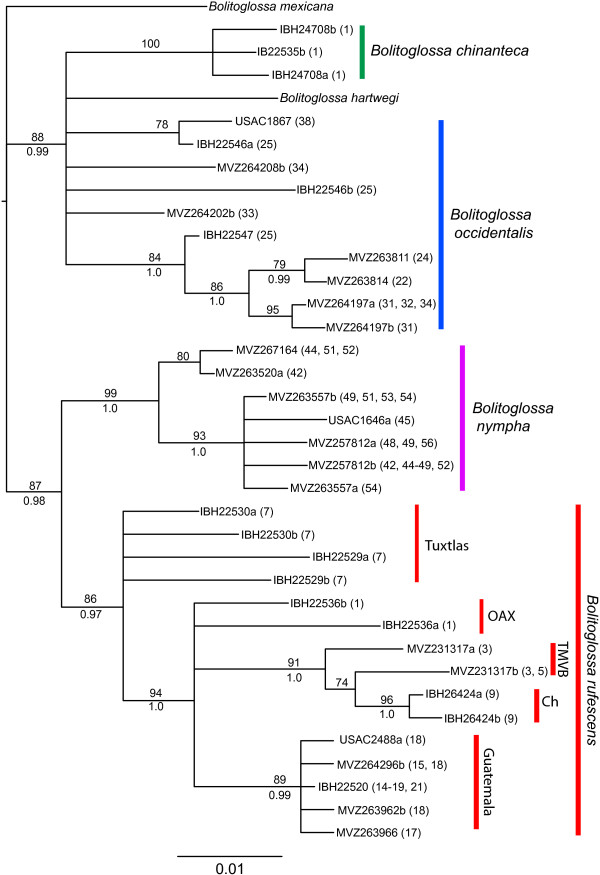
**Results of RAxML analysis of *POMC *sequences.** Haplotypes from heterozygous individuals are designated by a and b following the voucher number. Only one individual per haplotype is shown on the tree; thus, heterozygous individuals sharing haplotypes with other individuals do not appear. Numbers in parentheses after voucher numbers refer to all sampled localities (from Table 1) where haplotype was found. Bootstrap support values displayed above branches and posterior probabilities from Bayesian analysis below branches. Bootstrap values below 70 and posterior probabilities below 95 not shown. Geographic areas are shown for samples of *Bolitoglossa rufescens*. Abbreviations: Ch–Chiapas; TMVB–eastern terminus of Trans-Mexican Volcanic Belt; OAX–highlands of northern Oaxaca.

**Figure 4:**
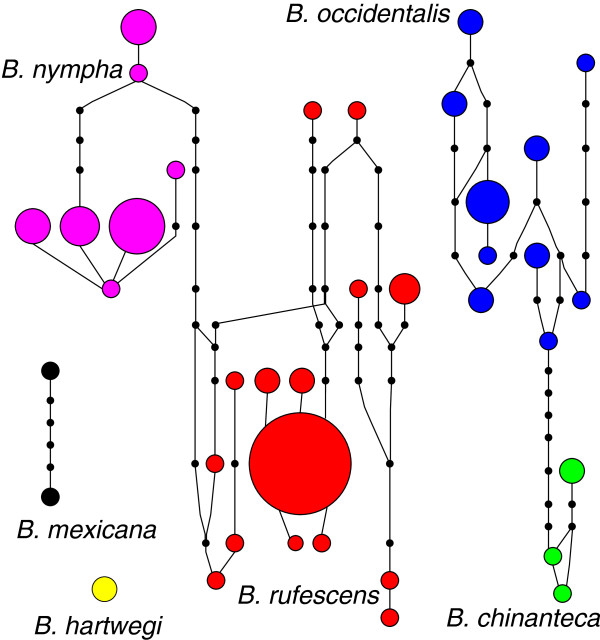
**Haplotype network for *POMC *data.** Haplotypes are colored by species: red, *B. rufescens*; blue, *B. occidentalis*; green, *B. chinanteca*; pink, *B. nympha*. Size of circles is proportional to haplotype frequency. Small black dots indicate unsampled haplotypes inferred from analysis.

Results from phylogenetic analyses of the *POMC* data (Figure 3) mirror those from mtDNA in most respects. *Bolitoglossa occidentalis* and *B. chinanteca* are again placed in a clade that is the sister lineage of the other two species of *Nanotriton*, and *B. chinanteca* is monophyletic. The sample from nearest the type locality of *B. bilineata* is again nested within a group of other samples of *B. occidentalis* from Chiapas, in agreement with the mtDNA results, providing no support for the distinctiveness of this named taxon. The single sample of *B. hartwegi* included in the dataset fell within this clade, rather than outside *Nanotriton*; this relationship is strongly supported (BS=88, PP=0.99), suggesting that the relationship between the subgenera *Nanotriton* and *Mayamandra* should be further investigated using additional markers. Both *B. rufescens* and *B. nympha* are supported as monophyletic. Within *B. rufescens*, the initial divergence is once again resolved to be between populations from Los Tuxtlas and all other populations (Figure 3), and samples on either side of the Isthmus of Tehuantepec are not reciprocally monophyletic. A haplotype network constructed using the program TCS with the POMC data shows four separate networks and similar patterns to the phylogenetic tree results (Figure 4). The first corresponds to *B. nympha* and *B. rufescens*, which are separated by 8 mutational steps. The second network consists of samples of *B. occidentalis* and *B. chinanteca*, which are separated by 7 mutational steps, and the final two networks correspond to samples of *B. hartwegi* and *B. mexicana* (the outgroup in phylogenetic analyses)*. Bolitoglossa rufescens* and *B. occidentalis* show both a higher diversity of haplotypes and more divergence between haplotypes compared to *B. nympha*. There was no discordance between the mitochondrial and *POMC* gene trees in terms of individuals being placed within clades corresponding to species; all samples from near contact zones fall into a clade corresponding to the same species in both the mtDNA and *POMC* gene trees. No individual has *POMC* haplotypes belonging to two different species, as we would expect if our dataset contained interspecific hybrids.

We are reluctant to undertake a molecular dating analysis of all divergences in the group due to a lack of appropriate fossil calibration points. Despite this, we estimated a chronogram with mtDNA data from all samples used in our phylogenetic analyses in order to calculate the divergence time of populations of *B. rufescens* that span the Isthmus of Tehuantepec. We used two different published substitution rate estimates for *cytb* from a fossil-calibrated phylogenetic analysis of plethodontids [36] and a rate that has been used as a general vertebrate molecular clock [37] in order to compare the timing of this divergence to that seen in other species. Populations of *B. rufescens* separated by the Isthmus of Tehuantepec were estimated to have diverged 3.3 million years ago (Ma) using the slower substitution rate estimate in the BEAST analysis, or 1.9 Ma using the faster rate.

**Figure 5:**
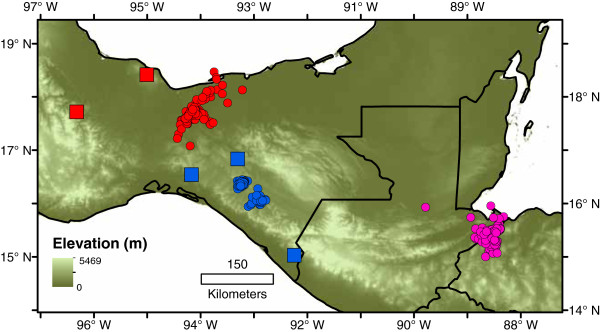
**Results of Phylomapper analyses.** Small circles indicate estimated location of ancestor of each clade from 100 replicate runs accounting for phylogenetic uncertainty in mtDNA gene tree. Squares indicate alternative ancestral locations used for hypothesis testing. Ancestral locations for *B. nympha* were not tested due to a lack of resolution in the mtDNA gene tree (see Methods). Red symbols: *Bolitoglossa rufescens*; blue symbols: *B. occidentalis*, pink symbols: *B. nympha*.

Results of analyses of phylogeographic structure and ancestral origin of clades from Phylomapper analyses indicate that significant phylogeographic structure is present within both *Bolitoglossa occidentalis* and *B. rufescens.* Both species show a significant association between phylogenetic and geographic distance, as measured by the scaled dispersal parameter (*Ψ*), compared to the null expectation derived from randomizing the location of sampled individuals [32] (*B. rufescens*: mean *Ψ*=177.66, p<0.0001; *B. occidentalis*: *Ψ*=33.84, p<0.0001). The ancestral location of *B. rufescens* was reconstructed as being on the eastern side of the Isthmus of Tehuantepec (Figure 5), but is not significantly different from the null expectation of an origin at the center of its range (*χ*^2^=1.69, p=0.43, df=2). Furthermore, likelihood ratio tests failed to reject an ancestral location of *B. rufescens* in either the northern highlands of Oaxaca (*χ*^2^=4.79, p=0.09, df=2) or in Los Tuxtlas (*χ*^2^=5.25, p=0.07, df=2). For *B. occidentalis*, the location of the ancestor was estimated to be on the eastern side of the Sierra Madre de Chiapas (Figure 5). The ML estimate of the location of the ancestor of *B. occidentalis* was significantly different from the center of the species’ range (*χ*^2^=6.20, p=0.045, df=2). Alternate locations for the ancestor of *B. occidentalis* in the Berriozabal area (localities 24), Sierra de los Chimalapas (locality 22), and the Pacific coast of southeastern Chiapas (locality 36) were all significantly less likely than the ML estimate (Berriozabal: *χ*^2^=10.08, p=0.0064; los Chimalapas: *χ*^2^=18.80, p<0.0001; Pacific coast: *χ*^2^=44.02, p<0.0001; df=2 for all tests).

**Figure 6:**
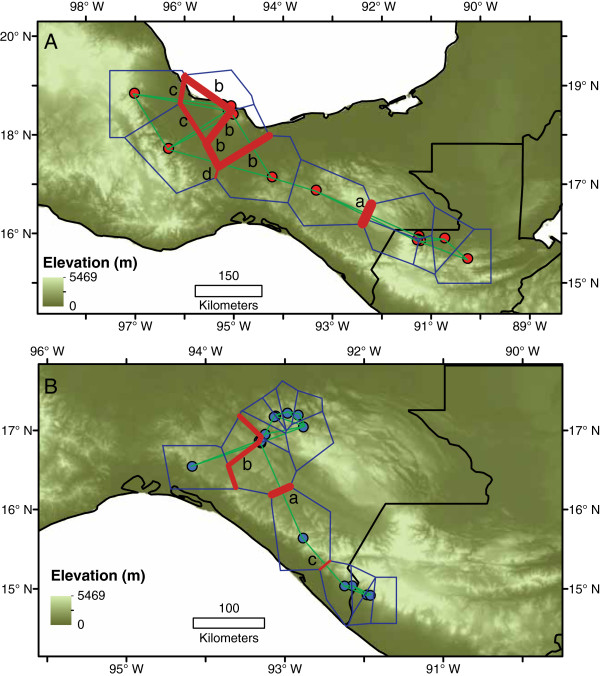
**Results of Barrier analyses for *B. rufescens *and *B. occidentalis*. A)** Four barriers between populations of *B. rufescens*, with Barrier a as the first barrier inferred and Barrier d as the last. Barrier a corresponds to a division between samples from Guatemala and all others; b separates some populations from Los Tuxtlas, c separates the remaining population from Los Tuxtlas, and d separates populations on either side of the Isthmus of Tehuantepec. **B)** For *B. occidentalis*, Barrier a separates populations from Guatemala and southeastern Chiapas from all others, Barrier b divides the Cerro Baul population and one population from the Berriozabal area from all others, and Barrier c separates populations from the Pacific coast of Guatemala and southeastern Chiapas from the single population in the El Triunfo area. Blue lines indicate polygons from Voronoi tessellation, green lines show Delaunay triangulation.

For species with significant phylogeographic structure detected in Phylomapper, we performed analyses to determine the geographic location of genetic barriers using the program Barrier v2.2. These analyses identified the split between populations in Guatemala from all others in Mexico as the primary genetic barrier within *B. rufescens*. Two barriers separating the Los Tuxtlas populations from others in Mexico were then demarcated, followed by a fourth barrier across the Isthmus of Tehuantepec (Figure 6). Within *B. occidentalis*, the first barrier identified was between a group of populations on the Pacific coast of Guatemala/southeastern Chiapas and the Sierra Madre de Chiapas (Localities 35–39) and all other populations in Mexico. The second barrier delineated the populations in the Sierra de los Chimalapas (Locality 22) and a single locality from central Chiapas from others in central and northern Chiapas, and the third separated the single population in the Sierra Madre de Chiapas (Locality 35) from others in southeastern Chiapas and Guatemala.

A hierarchical Analysis of Molecular Variance (AMOVA) for *Bolitoglossa rufescens* was used to test for significant genetic structure at two levels: 1) among regional groups consisting of Los Tuxtlas, Nuclear Central America, and the TMVB/Oaxacan highlands, and 2) among populations from Chiapas and Guatemala within Nuclear Central America and from the TMVB and Oaxacan highlands within third group. The AMOVA showed significantly more genetic variance partitioned between populations within regional groups compared to the null expectation from permuting haplotypes among populations within groups (sum of squared deviations [SSD] among populations within groups=81.50, total SSD= 266.68, DF=2. p<0.0001, DF=2. p<0.0001), but variance among regional groups was not larger than would be expected by chance (SSD among groups=125.18, total SSD = 266.68, DF=2. P=0.60). An AMOVA for *B. occidentalis* with four populations from the Sierra de los Chimalapas, northern and central Chiapas, the Sierra Madre de Chiapas, and the Pacific coast of southeastern Chiapas and Guatemala showed significantly more genetic variance partitioned among populations than expected by chance (SSD among populations= 97.419, total SSD= 157.813, DF=3, p<0.0001).

## Discussion

A high degree of phylogeographic structure characterizes both *Bolitoglossa rufescens* and *B. occidentalis*, as revealed both by the significant phylogeographic association test from Phylomapper and the presence of multiple divergent lineages present within both species. *Bolitoglossa rufescens* is known from a number of localities on both sides of the Isthmus of Tehuantepec and in the isolated Sierra de los Tuxtlas, making it one of only two species of salamander (along with *B. platydactyla*) to be distributed in all three of these areas. Although the Phylomapper results reconstruct a Central American origin for the species, an ancestral location within either the highlands of northern Oaxaca or in Los Tuxtlas could not be rejected. Indeed, the null hypothesis of an ancestral location at the center of the species' current range could also not be rejected. Our inability to distinguish between these hypotheses may be due to the fact that the three major areas where *B. rufescens* is found meet in approximately the center of its range, and its history appears to be relatively deep in both Los Tuxtlas and Nuclear Central America. Nucleotide diversity for *16S* is low in the TMVB/northern Oaxacan highlands (*π=*0.0040 ± 0.0037) compared to both los Tuxtlas (*π=*0.014 ± 0.011) and Central America (*π=*0.021 ± 0.012), which points to an origin for the species outside of the TMVB/northern Oaxacan highlands. The fact that initial divergences within *B. rufescens* are between Los Tuxtlas and all other areas in both the mtDNA and *POMC* gene trees suggests that this area may have been the origin of the species, or minimally that *B. rufescens* has been present in Los Tuxtlas for a long period of time. Volcanism that created the Sierra de los Tuxtlas began around 7 Ma, and several of the major volcanoes were formed sometime between from 3–1 Ma [38]. Although no calibration points are available for an accurate molecular dating analysis, the deep divergence between the samples from Los Tuxtlas and the clade from the TMVB/Oaxacan highlands and Nuclear Central America for both mtDNA and *POMC* most likely coincided with the early formation of the Tuxtlas volcanic complex.

Multiple species of amphibians show either disjunct distributions (*Pseudoeurycea werleri*[17], *Pseudoeurycea nigromaculata*[39], *Anotheca spinosa*[40]) or sister species pairs (*Pseudoeurycea orchimelas* and *P. orchileucos*[41]; *Thorius pennatulus* and *T. narismagnus*[16,39]) between the eastern terminus of the TMVB of Veracruz or highlands of northern Oaxaca and Los Tuxtlas, while other sister species pairs are found on either side of the Isthmus of Tehuantepec in Los Tuxtlas and the Sierra de los Chimalapas [42]. The only other lowland *Bolitoglossa* in the area, *B. platydactyla*, is present at low elevations in the Sierra Madre Oriental, eastern terminus of the TMVB, northern Oaxaca, Los Tuxtlas, and in northern Chiapas. Campbell (1984) proposed a closer relationship between the herpetofauna of Los Tuxtlas and the Chimalapas than between Los Tuxtlas and the northern Oaxacan highlands, and observed that the lowland area between los Tuxtlas and other highland areas of southern Mexico must have contained forest in the past to allow for interchange between these areas. The lowland areas around Los Tuxtlas today, which include both savannah and wetlands, do not contain any known populations of *B. rufescens* that would indicate a more continuous distribution in the recent past between these volcanoes and other areas where *Nanotriton* are found, although more searches would be needed to confirm this. The high nucleotide diversity of Los Tuxtlas compared to the TMVB/northern Oaxacan highlands and the high genetic distance between Los Tuxtlas and all other populations of *B. rufescens* follow the pattern of divergence and endemism seen in more highland amphibian and reptile species with similar distributions and indicate the Los Tuxtlas populations have been isolated from other highland areas of southern Mexico for a long time. Indeed, the *B. rufescens* from Los Tuxtlas are the only population of the species with maxillary teeth [29], a morphological character often used to delimit species of tropical salamanders [16,43]. The Los Tuxtlas populations may warrant description as an additional species endemic to the volcanic complex, and merit further study.

Mulcahy et al. [44] found that the Isthmus of Tehuantepec corresponded to a phylogeographic break in both *Incilius valliceps* and *Rhinella marina*, two lowland toads distributed widely in Mexico and Central America. They dated this divergence to approximately 2–3 Ma in both species, and suggested that these data were consistent with the existence of a Pliocene seaway across the Isthmus that may have caused vicariance between populations on either side. Using divergence values recalculated separately for each gene, their data show levels of divergence across the Isthmus for both species that are higher those seen for *B. rufescens* for *16S* (*R. marina*: mean *16S* GTR distance=0.011, *I. valliceps*: 0.0088) but lower for *cytb* (*R. marina*: mean *cytb* GTR distance=0.026; *I. valliceps*=0.020); *B. rufescens* populations from the TMVB/northern Oaxacan highlands and those from Chiapas in Nuclear Central America have mean pairwise GTR distances of 0.0062 for *16S* and 0.040 for *cytb*. The fact that the trans-Isthmus divergence values for *B. rufescens* are not consistently higher or lower suggests that *B. rufescens* may have been affected by this barrier in a similar manner as the toads. Although substitution rate estimates for this group of tropical salamanders are not available, using two substitution rates for *cytb* from the literature gives a divergence estimate of 1.9 or 3.2 Ma, roughly concordant with the timing of the Pliocene seaway; the second estimate uses a rate derived from a phylogenetic analysis of plethodontids [36], and may represent a better estimate of the divergence time. Samples of *B. rufescens* from north and south of the Isthmus are not reciprocally monophyletic for mtDNA; samples from the TMVB/northern Oaxacan highlands render those from Central American paraphyletic. This suggests that any phylogeographic structure caused by the breaks in forested habitat associated with formation of the Isthmus or by formation of a seaway across the Isthmus postdates earlier phylogeographic structure associated with geologic or climatic barriers within Central America. The fact that genetic structure within *B. rufescens* associated with the Isthmus is relatively shallow compared to that within Nuclear Central America is reflected in the Barrier analysis results, which showed that the Isthmus corresponds to the fourth most important barrier within the species, after barriers within Nuclear Central America and Los Tuxtlas. Similarly, the AMOVA results show that while significant genetic structure is present among populations within regions, genetic structure is not significant at the regional level, indicating that divisions between regions such as the Isthmus do not correspond to locations of the primary genetic divisions between the species.

In addition to its possible role as a barrier between the Oaxacan highlands and Central America, the Isthmus may have isolated populations in Los Tuxtlas from those in Central America. The much deeper divergence seen between Los Tuxtlas and all populations in Central America may have been partly driven or reinforced by the formation of the Isthmus in the late Miocene or early Pliocene [45]. At an even deeper timescale, *Bolitoglossa occidentalis* and *B. chinanteca* were found to be sister taxa and are separated by the Isthmus of Tehuantepec. Based on our rate calibrations, their divergence is much too old to have been caused by a Pliocene seaway and likely predates the event. Given this difference in timing of the divergence between the two species and the formation of the Isthmus, the fact that their ranges are currently separated by the Isthmus suggests that caution must be used when inferring a causal role for geographic barriers based solely on overlap with genetic breaks.

Despite its small range and its distribution entirely within the Mayan geological block [46] of Nuclear Central America, *B. occidentalis* displays a high level of genetic diversity for both mtDNA and *POMC*. The Phylomapper results show significant phylogeographic structure within the species and reject an origin in any of the three main clusters of populations within our sampling or from the center of its current distribution. These results provide strong support for an origin of *B. occidentalis* on the eastern side of the Sierra Madre de Chiapas, near one of our sampled populations (Locality 35). While increased sampling of populations on both sides of the Sierra Madre de Chiapas could potentially change this result, our analyses indicate that *B. occidentalis* arose in an area of Caribbean drainage. *Bolitoglossa rufescens* is entirely confined to Caribbean-draining areas of Mexico and Central America, while *B. occidentalis* is primarily found on the Pacific side of Central America [15] (Figure 1). If both species arose within the Caribbean drainage, some other factor besides isolation across the Caribbean-Pacific divide must have been responsible for their initial divergence. The Barrier results show that the primary division within the species is between populations in southeastern Chiapas and Guatemala and all others, and the AMOVA results also show significant structure between the different areas where *B. occidentalis* is found.

The limited divergence between populations and lack of strong phylogeographic structure within *Bolitoglossa nympha* stands in contrast to that seen in both *B. rufescens* and *B. occidentalis*. *Bolitoglossa nympha* ranges across a major geographic barrier, the Motagua-Polochic fault system [21,22], yet this fault and associated subhumid areas seem not to have caused vicariance or population isolation within *B. nympha*. *Bolitoglossa nympha* and *B. rufescens* may have been separated by the Motagua-Polochic fault in the past, with a subsequent expansion of *B. nympha* to the northwest of the fault, bringing the two species into secondary contact. Likewise, areas where the distributions of *B. nympha* and *B. rufescens* approach each other in both the Sierra de los Cuchumatanes (localities 14–18 and 40, Figure 1C) and the Sierra de Xucaneb (localities 21 and 41–42; Figure 1D) in Guatemala present no obvious geological or climatic barrier that could have either caused the divergence between the two species or maintained them in allopatry. The low haplotype diversity and lack of strong phylogeographic structure within *B. nympha* suggest that the species may have undergone a historical bottleneck or reduction in range. Modeling of the extent of wet forest habitat, with which species of *Nanotriton* are generally associated, in Central America at the Last Glacial Maximum predicted an absence of this habitat in northeastern Honduras and eastern Guatemala [47], covering most of the current range of *B. nympha.* While this area was previously hypothesized as a Pleistocene refugium for plant taxa [48], the absence of humid forest over most of the range of *B. nympha* east of the Motagua fault during the Pleistocene could explain both the lack of strong phylogeographic structure within the species compared to *B. rufescens* and *B. occidentalis* and the lack of a phylogeographic break across the Motagua-Polochic fault.

## Conclusions

Species of *Nanotriton* have a long history in Central America. The deepest divergences within and between species of *Nanotriton* do not appear to have been caused by restricted dispersal across biogeographic barriers identified for other taxa, such as the Isthmus of Tehuantepec and Motagua-Polochic fault system. While the Isthmus of Tehuantepec did cause vicariance of populations of *Bolitoglossa rufescens*, this divergence is shallow compared to others within both *B. rufescens* and *B. occidentalis*. The distinctiveness of *B. rufescens* from Los Tuxtlas appears to be the exception to this pattern, and reinforces the importance of Los Tuxtlas as a site of high endemism and biogeographic distinctiveness within southeastern Mexico. Aside from Los Tuxtlas, the highest intrapopulation divergence within both *B. occidentalis* and *B. rufescens* has taken place within the Mayan block of Nuclear Central America. This relatively strong phylogeographic structure over small spatial scales could result in the formation of separate species over longer timescales. The external morphological similarity of all species in the subgenus has hidden the high phylogeographic structure present within two of these species, and subsequent morphological examination of populations from Los Tuxtlas or *B. occidentalis* from Chiapas could show that additional independent lineages warrant recognition as distinct species. Despite their morphological similarity, we found no evidence of introgression at or near contact zones. Differences in levels of phylogeographic structure between *B. rufescens* and *B. nympha* may be related to regional differences in historical forest extent, which is hypothesized to have varied over time [47,48]. This phenomenon should be tested by examining patterns of genetic variation within other forest-inhabiting taxa with similar distributions to these two salamander species. The phylogeographic history of *Nanotriton* serves as a useful comparison to both higher elevation forest taxa [21,22,49] and lowland, arid-adapted species [24], and provides an important addition to our understanding of factors responsible for population divergence and speciation across a geologically complex landscape.

## Methods

### Study system and sample collection

Populations of all four species of *Nanotriton* were sampled throughout Mexico, Guatemala and Honduras during fieldwork from 2005 to 2011, as well as from the Museum of Vertebrate Zoology tissue collection. Populations of *B. rufescens* in eastern Guatemala and Honduras belong to a second, recently described species, *B. nympha*[35]; this species was described only from the type locality, but other populations from the region bear a morphological resemblance to the type series [27,29]. *Bolitoglossa occidentalis* occurs from extreme eastern Oaxaca to the Pacific coast of Guatemala; we examined a single individual reported to *B. occidentalis* from the Caribbean side of Honduras [50], and determined it to be a juvenile of another subgenus (most likely subgenus *Bolitoglossa*). When this record is excluded, nearly all portions of the known range of these species were sampled in our study. Permits for specimen collection and export were provided to GPO in Mexico by SEMARNAT, to CRVA in Guatemala by CONAP, and to SMR in Honduras by COHDEFOR.

We collected a total of 96 individuals from 57 localities (Additional file 4: Table S1, Figure 1). Most individuals were found by searching in the outer layers of the trunks of banana plants in plantations or coffee groves, but some were found in arboreal bromeliads, under cover objects, or at night on vegetation. Liver and/or tail tissue was collected and stored either in ethanol, liquid nitrogen, or RNALater buffer in the field, and subsequently transferred to −80°C. Voucher specimens were deposited in the Museum of Vertebrate Zoology (MVZ) at the University of California, Berkeley, the Instituto de Biología, Universidad Nacional Autónoma de México (IBH), or the Museo de Historia Natural, Universidad de San Carlos, Guatemala (USAC).

### Sequencing and phylogenetic analysis

We extracted DNA from liver or tail tissue using either DNeasy extraction kits (Qiagen, Valencia, CA, USA) or a guanidine thiocyanate extraction protocol. The guanidine thiocyanate protocol involves cell lysis at 55°C for 3–12 hours, protein precipitation using guanidine thiocyanate followed by centrifugation for 10 min, and DNA precipitation using 100% isopropanol followed by centrifugation for 10 min. We sequenced two mitochondrial genes, the large subunit ribosomal RNA gene (*16S*) using primers 16Sar and 16Sb [51] and cytochrome *b* (*cytb*) using primers MVZ15 and MVZ16 [52], as well as one nuclear gene, proopiomelanocortin (*POMC*) using primers POMC_DRV_F1 and POMC_DRV_R1 [53]. PCR amplification consisted of an initial denaturation step at 95°C for 2 min, followed by 38 cycles of denaturation at 95°C for 30 s, annealing at 48°C (*16S* and *cytb*) or 57°C (*POMC*) for 1 min, and extension at 72°C for 1 min, with a final extension at 72°C for 7 min. PCR products were purified using 1uL of EXOSAP-IT (USB Corp., Cleveland, OH, USA), cycle sequenced with BigDye3.1 terminator sequencing (Applied Biosystems, Foster City, CA, USA), purified using ethanol precipitation and run on an ABI-3730 capillary sequencer (Applied Biosystems, Foster City, CA, USA). Sequences were edited using Sequencher (GeneCodes, Ann Arbor, MI, USA) or Geneious v 5.1.7 [54] GenBank accession numbers for all sequences are given in Additional file 4: Table S1.

Sequences were aligned with Muscle v3.6 [55] using default parameters. Alignment lengths were 522 base pairs (bp) for *16S*, 772 bp for *cytb*, and 481 bp for *POMC*. Gametic phase of *POMC* sequences was determined computationally using PHASE [56], and haplotype determinations from the best pairs output were used. When an individual had two distinct haplotypes for *POMC*, both were used in gene tree reconstruction. We constructed gene trees separately for concatenated mitochondrial genes (*16S*+*cytb*) and *POMC* using both maximum likelihood (ML) and Bayesian analyses, and removed redundant haplotypes prior to phylogenetic analysis. For ML analyses, the program RAxML v7.0.4 [57] was used. Mitochondrial data were partitioned by gene, and *cytb* data were further partitioned by codon position. A GTR+G+I substitution model was used for all ML analyses, and 1000 bootstrap replicates were run to assess nodal support; models less complex than the GTR model are not implemented in RAxML. We estimated gene trees using MrBayes 3.1.2 [58], with two runs and four chains (one cold, three heated) per run. MCMC analyses were run for 2*10^7^ generations, sampled every 1000 generations, and the first 5000 samples were discarded as burn-in. Rate variation across partitions was permitted, and default priors were used for other parameters. We used the sliding window and compare plots in the program AWTY [59] to check for convergence in Bayesian analyses. A sequence of *Bolitoglossa* (*Bolitoglossa*) *mexicana* was used as the outgroup for all phylogenetic analysis, and a sequence of *Bolitoglossa* (*Mayamandra*) *hartwegi* was also included in the dataset, given that the subgenera *Bolitoglossa* and *Mayamandra* were found to be the closest relatives of *Nanotriton* in previous analyses of mtDNA [20]. Sequence alignments and Bayesian consensus trees are archived in TreeBase (Submission 13756; http://purl.org/phylo/treebase/phylows/study/TB2: S13756).

For Bayesian analyses, we tested several partitioning strategies for the mtDNA dataset: 1) all data as a single partition, 2) *16S* and *cytb* as separate partitions (two partitions), and 3) *16S* as one partition with *cytb* further partitioned by codon position (four partitions). For *POMC*, we compared results with all data as single partition and with each codon position as a separate partition. The program MrModeltest [60] was used to determine the most appropriate model of nucleotide substitution for each partition using the AIC. We determined the most appropriate partitioning strategy for each locus using Bayes factors [61]. Comparison of results of Bayesian analyses with different partitioning strategies supported the 4-partition strategy for mtDNA (2ln(Bayes factor) 4 vs. 2 partitions–466; 4 vs. 1 partition–624; 2 vs. 1 partition–158) and 3 partitions for the *POMC* data (2ln(Bayes factor) 3 vs. 1 partition–15). The following substitution models were used in the favored partitioning strategy: *16S*,– GTR+I+G; *cytb* codon positon 1, 2 – HKY+G; *cytb* codon position 3, *POMC* codon position 3 – GTR+G; *POMC* codon position 1 – F81; *POMC* codon position 2 – HKY+I. A haplotype network for the *POMC* data was constructed using TCS v 1.2.1 [62].

### Detection of interspecific hybridization

No examples of hybridization are currently known for the subgenus *Nanotriton*, and these phylogeographic data are suitable for assessing whether these morphologically similar species come into contact and hybridize. In order to detect possible interspecific hybrids, larger numbers of salamanders were sequenced for zones where different species may contact each other in Chiapas, Mexico and Guatemala. Although *B. chinanteca* and *B. rufescens* are known to occur syntopically in Oaxaca, Mexico [27], only a few samples from this locality were available. In the area of Berriozabal, Chiapas, Mexico (localities 13, 23–25; Figure 1) and Chancolín, Guatemala (localities 14–18, 40; Figure 1), multiple individuals per locality were included in both *16S* and *POMC* phylogenetic analyses. Not all these individuals were sequenced for *cytb*, since both mitochondrial genes are inherited as a single unit. If F1 hybrids were included in the sample, we would expect to see some individuals with distinct *POMC* haplotypes from two different species, given the degree of structure in the *POMC* gene tree (see Results). Additionally, for both F1 hybrids and some backcrosses, we would expect to see individuals with *16S* haplotypes from one species and *POMC* haplotypes from a different species.

### Phylogeographic analyses

In order to determine the geographic origin of the three species with larger distributions (all except *B. chinanteca*), we used Phylomapper v1 [32]. This program uses geographic coordinates of collection localities of samples included in a phylogeny, along with branch length information, to estimate the geographic location of the ancestor of a clade using a random walk model of migration. Because branches of zero length produce infinite dispersal distance estimates (Phylomapper manual), the number of identical haplotypes was first reduced by sampling only a single individual from each site or cluster of sites. Mitochondrial sequences (either *16S* and *cytb* or *16S* alone) were used to construct a mtDNA gene tree using MrBayes from this sample set, with the same analysis parameters as in the analysis of all mtDNA sequences. To deal with remaining redundant haplotypes, one tree from the 15,000 fully resolved trees sampled from the posterior distribution by MrBayes was selected for each run using the “random” option in Phylomapper. The geographic origin of each species was calculated separately, using 100 runs of Phylomapper. For each analysis, rate smoothing was conducted for the clade of interest (the species whose geographic origin was being estimated), rather than for the entire *Nanotriton* clade.

Phylomapper was also used to test statistically for phylogeographic association, or a correlation between geographic distance and genetic distance, within a clade and for a difference between the estimated location of the ancestor of a clade and the geographic center of that clade. The Bayesian consensus tree was used for all hypothesis testing, rather than choosing a tree randomly from the posterior distribution, in order to conduct all tests on the same gene tree. Polytomies in the consensus tree were resolved arbitrarily by inserting branches of very short length (0.0001). Statistical testing was not done for *B. nympha* because resolving the large number of polytomies between closely related haplotypes significantly changed the likelihood compared to runs using randomly chosen trees from the posterior distribution. For the phylogeographic association test, geographic coordinates from sampling localities were assigned randomly across tips and 10000 iterations were used to construct a null distribution of the scaled dispersal parameter (*Ψ*). Several additional hypotheses related to the ancestral location of clades were also tested by fixing the location of the ancestor of the clade, optimizing the other parameters in the model and comparing the likelihood of the constrained model (location fixed) with the model with the ancestral location unconstrained using a likelihood ratio test with two degrees of freedom [32]. Within each region tested as an alternative ancestral location, the geographically closest population in our dataset to the estimated origin of the clade was used as the point locality for the alternative origin location. *Bolitoglossa rufescens* was tested for an origin in the northern Oaxacan highlands (locality 1), and for an origin in the Los Tuxtlas region of Veracruz (locality 8). *Bolitoglossa occidentalis* is confined to the Nuclear Central America region, but is distributed primarily in three areas within the region, and was tested for an ancestral location in each: north-central Chiapas (locality 24), the Pacific coast (locality 36), and the Sierra de los Chimalapas (locality 22).

For species with a significant result from the Phylomapper phylogeographic association test, we used the program Barrier v2.2 [63] to infer the geographic location of barriers between samples with Monmonier’s (1973) maximum difference algorithm [64]. Barrier identifies the spatial location of genetic breaks by dividing up geographic space using a Voronoi tessellation, creating a network of polygons each edge of which is equidistant to two sampling localities, and the centroids of these polygons (sample localities) are connected in a Delaunay triangluation, Monmonier’s algorithm finds the edge of the triangulation with the maximum genetic distance, and traces a barrier along the edge of the Voronoi polygon perpendicular to this edge. It proceeds along adjacent edges until the edge of the triangulation or a previously defined barrier is reached [63]. We used PAUP [65] to calculate GTR distances between samples used in the Phylomapper analyses, and ran the analysis with four barriers for *B. rufescens* and three for *B. occidentalis* (which has a smaller geographic range). Larger numbers of barriers were tested, but began to finely subdivide geographically proximate populations.

We used Arlequin v3.5 [66] to calculate nucleotide diversity for clades within species with the Tamura-Nei (TN) [67] substitution model (the most parameterized model implemented in Arlequin) with a gamma correction, in order to compare levels of genetic diversity within subregions. We used the same mtDNA dataset in Phylomapper in order to remove multiple redundant haplotypes from sites near contact zones. The value of gamma from the substitution model chosen by MrModeltest2.2 was used for distance calculation. For *Bolitoglossa rufescens*, samples were grouped into those from the eastern terminus of the TMVB/northern highlands of Oaxaca, Mexico (localities 1–5), Central America (localities 7–13), and those from Los Tuxtlas (localities 6–8), and the first two populations were used to compare divergence levels across the Isthmus of Tehuantepec. We also calculated nucleotide diversity for each species as a whole. Finally, we calculated mean GTR distances between species, as well as between geographic groups of populations within species of *Nanotriton*, using PAUP [65]. We also recalculated GTR distances across the Isthmus for two toad species (*Rhinella marina* and *I. valliceps*) from Mulcahy et al. [44], separating their *16S* and *cytb* data in order to allow comparison with our data. We also performed a hierarchical Analysis of Molecular Variance (AMOVA) [68] in Arlequin for *B. rufescens* and *B. occidentalis*, which showed significant phylogeographic structure in the Phylomapper analyses. Populations were divided into three regional groups for *B. rufescens*: Los Tuxtlas, Nuclear Central America, and the TMVB/Oaxaca Highlands, and the latter two groups were subdivided into populations from Chiapas, Guatemala, TMVB, and Oaxacan highlands. A hierarchical AMOVA was performed using *16S* sequences, with 1000 permutations used to assess significance. For *B. occidentalis*, an AMOVA with four groups was performed: the Sierra de los Chimalapas (locality 22), populations from northern and central Chiapas (localities 23–34), the population from the Sierra Madre de Chiapas (locality 35), and populations from the Pacific coast of southeastern Chiapas and Guatemala (localities 36–39).

### Divergence dating

No fossil calibration points are available for tropical salamanders, complicating the use of molecular data to infer divergence dates. In order to gain a rough estimate of the divergence time between populations of *B. rufescens* on either side of the Isthmus of Tehuantepec, we used two different substitution rate estimates for *cytb* in a BEAST analysis [69] of the mtDNA data. The first rate of 0.0062 substitutions/site/Myr per lineage was estimated using a variety of fossil calibration points on a mitochondrial phylogeny of the Plethodontidae [36], while the second, faster rate of 0.01 substitutions/site/Myr [37] has been used as a general vertebrate mtDNA clock. The BEAST analysis was done a separate GTR+G substitution model for gene. An uncorrelated lognormal relaxed clock model was used to estimate divergence dates. Analyses were run for 20 × 10^9^ generations, sampled every 1000 generations, and Tracer v1.6 [70] was used to summarize posterior distributions of divergence times.

## Competing interests

The authors declare that they have no competing interests.

## Authors’ contributions

SMR helped design study, conducted fieldwork, carried out the molecular genetic studies, performed phylogenetic analyses, and drafted the manuscript. GPO and DBW helped design study, carried out fieldwork, and assisted with manuscript preparation. CRVA and RLR conducted fieldwork and assisted with manuscript preparation. All authors read and approved the final manuscript.

## Supplementary Material

Additional file 1 **Table S2.** General time reversible (GTR) distances between *16S* haplotypes used in phylogenetic analyses. Click here for file

Additional file 2 **Table S3.** General time reversible (GTR) distances between *cytb* haplotypes used in phylogenetic analyses. Click here for file

Additional file 3 **Table S4.** General time reversible (GTR) distances between *POMC* haplotypes used in phylogenetic analyses. Click here for file

Additional file 4 **Table S1.** Museum catalog numbers, locality information, geographic coordinates, and GenBank accession numbers for all tissues used in phylogenetic analyses. Click here for file
